# Examining sea levels forecasting using autoregressive and prophet models

**DOI:** 10.1038/s41598-024-65184-0

**Published:** 2024-06-21

**Authors:** Leena Elneel, M. Sami Zitouni, Husameldin Mukhtar, Hussain Al-Ahmad

**Affiliations:** College of Engineering and Information Technology, University of Dubai, Dubai, United Arab Emirates

**Keywords:** Sea level rise, Climate change, Autoregressive models, Prophet model, Projection and prediction, Scientific data

## Abstract

Global climate change in recent years has resulted in significant changes in sea levels at both global and local scales. Various oceanic and climatic factors play direct and indirect roles in influencing sea level changes, such as temperature, ocean heat, and Greenhouse gases (GHG) emissions. This study examined time series analysis models, specifically Autoregressive Moving Average (ARIMA) and Facebook’s prophet, in forecasting the Global Mean Sea Level (GMSL). Additionally, Vector Autoregressive (VAR) model was utilized to investigate the influence of selected oceanic and climatic factors contributing to sea level rise, including ocean heat, air temperature, and GHG emissions. Moreover, the models were applied to regional sea level data from the Arabian Gulf, which experienced higher fluctuations compared to GMSL. Results showed the capability of autoregressive models in long-term forecasting, while the Prophet model excelled in capturing trends and patterns in the time series over extended periods of time.

## Introduction

Climate change poses one of the most formidable challenges in the 21st century, inducing profound global transformations. The escalating levels of GHG can be observed through increasing temperatures and melting ice sheets, which resulted in an increasing GMSL that exceeded historical observations^1^. Over the past century, GMSL has risen by over 3 mm per year, and it is projected to reach a rate of 0.77 m by the end of the century^2^. The impact of GMSL has resulted in far-reaching consequences across various aspects including socioeconomic, biophysical, and environmental aspects^1^. Coastal erosion rates have risen in a significant number of coastal regions globally. The occurrence and severity of flooding and other extreme weather events have notably heightened in low-lying land and other vulnerable locations, particularly in Southeast Asian nations^3^ and Small Islands Developing States^4^. Other climatic factors, such as El Niño oscillations, contribute to this issue and amplify the challenges faced in these areas^5^. Moreover, the rise in GMSL and its consequential physical effects pose substantial risks to economic activities, human settlements, and ecosystems in coastal regions.

Historical observations of oceanic and climatic factors that contribute to sea level changes are utilized in modeling and analysis approaches. Over the past few years, machine learning and deep learning techniques have been widely used to analyze and forecast sea level variations at different spatial scopes^2^. Seasonal time series data of the years between 1993 and 2018 in the Bay of Bengal, were utilized to predict GMSL variation with respect to Relative Mean Sea Level (RMSL) using autoregressive models^6^. A three-step analysis pipeline was used by^7^, encompassing (1) dynamic structural equation modeling via vector autoregressive structural equation modeling (VAR-SEM) to unveil the interrelation between the time series of different contributing factors to GMSL, (2) VAR to predict changes based on the interrelation among the contributing factors and GMSL, and (3) a comparative analysis study on forecasting seasonal GMSL that showed that models using moving average (MA) components provide better results than linear regression models^8^. The linear regression model was also found suitable for capturing sea level trends based on historical observations of GMSL^9^. Elneel et al.^10^ compared the influence of forecasting GMSL under the impact of additive water mass resulting from Antarctica ice melt, using univariate and multivariate autoregressive models. Tur et al.^11^ used linear and non-linear models to predict short-term sea level changes under the influence of air’s temperature and pressure. The Work of Balogun and Adebisi^12^ provided further insights on the influence of different contributing factors in predicting sea level changes. Their work included categorizing the contributing factors to sea level variation into oceanic and climatic, in order to examine the significance of using either or both categories in predicting local sea level changes at different locations in Malaysia. VAR and ARIMA prediction models were tested and results showed that using both categories enhances the prediction results while ARIMA will perform better in locations where tide influence is higher than the one caused by oceanic-climatic factors. Long-term forecasting showed that the increasing trend of sea levels will continue but without acceleration^13^. Other models such as exponential Gaussian Process Regression (GPR) were also found effective in capturing short-term forecasting of changes in water level compared to other regression models^14^. Deep learning models also exhibited good performance in forecasting sea level forecasting under certain data requirements availability^8^.

The predictive analysis in recent research studies has utilized Facebook’s Prophet model. While it has not yet been utilized in sea level forecasting, based on our findings, it has been utilized in forecasting analysis of other climatological aspects. For instance, the Prophet model was employed to forecast seasonal changes resulting from climate change in water temperature and turbidity in the Ganga River, where it demonstrated better results compared to the Seasonal ARIMA model^15^. Prophet Model has also been recently used in temperature forecasting in the past few years^16–19^. It demonstrated efficiency in required computational time compared to other models and showcased its capabilities in handling complex seasonality patterns, interannual trends, temporal variations, long-term seasonality forecasting, and anomaly detection. Other applications of the Prophet model in climatological aspects include drought^20^, and changes in groundwater^21^. Given the current challenges posed by climatic data exhibits, including missing data, inconsistency, and unusual seasonality patterns, the Prophet model offers promising performance in this field (see “Facebook’s prophet model” section for more details).

**Figure 1:**
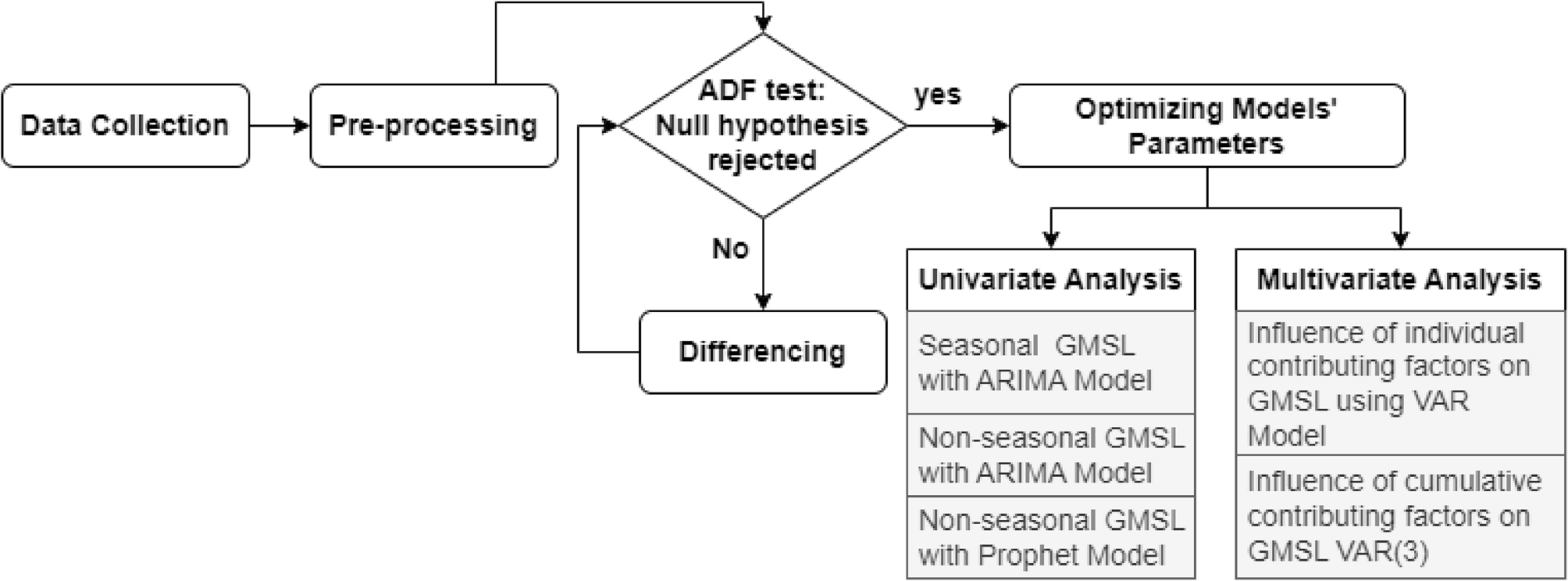
Methodology of comparative analysis of employing autoregressive models in forecasting sea level variations.

In this study autoregressive models were utilized for forecasting GMSL based on previous observations, encompassing seasonal and non-seasonal (annual) satellite altimetry data. In the context of this paper, yearly average data will be referred to as non-seasonal, and monthly annual data will be referred to as seasonal data. The application of the Prophet model in forecasting sea levels was also explored and compared to the output of autoregressive models. The investigation was further extended to assess the influence of different contributing factors on GMSL forecasting, including temperature, ocean heat, emissions of carbon dioxide (CO_2_), and methane (CH_4_). Figure 1 illustrates the used methodology in this work. Additionally, the influence of GMSL on RMSL are examined within the selected study area, namely the Arabian Gulf. The Arabian Gulf is a semi-enclosed sea and is classified as one of the warmest regions in terms of both water and air temperatures^22^. Due to the geographical distance from Greenland and Antarctica, the Arabian Gulf is not directly affected by the additional water mass resulting from glaciers and ice sheet melts. Hence, its water budget relies on the inflow of water from the Sea of Oman due to evaporation, as well as input water from rivers^23^. Despite this, The Arabian Gulf exhibits higher sea level fluctuations than GMSL. With a high population density residing in coastal areas across the Arabian Gulf, an increase in RMSL will impose high risks on coastal infrastructure and communities^24^.

## Results and discussion

### Univariate time series analysis

Two approaches were employed to assess the univariate time series of GMSL. In the first scenario, the p, d, and q parameters representing ARIMA model components (see section “Autoregressive models” for more details) were set to (2, 1, 3) and applied to the seasonal GMSL time series. This indicates that 2 lagged observations (p) were used, a first-order differencing (d) was applied to ensure the stationarity requirement of the input data, and a moving average window of size 3 (q) was used. Equation (1) represents the ARIMA (2,1,3) model formula.

1 $$\begin{aligned} \displaystyle \left( 1-\phi _1 B-\phi _2 B^2\right) (1-B)\left( y'_t-\mu \right) = \left( 1+ \theta _1 B+\theta _2 B^2+\theta _3 B^3\right) \ \varepsilon _t \end{aligned}$$

where *B* is the backshift operator, representing the lag of the time series; ${y'_t}$ is the differenced time series; ${\mu }$ is the mean of the differenced time series; ${\phi _1, \phi _2}$ are the Autoregressive AR coefficients; ${\theta _1, \theta _2, \theta _3}$ are the MA coefficients; ${\varepsilon _t}$ is the white noise error term at time t.

**Figure 2:**
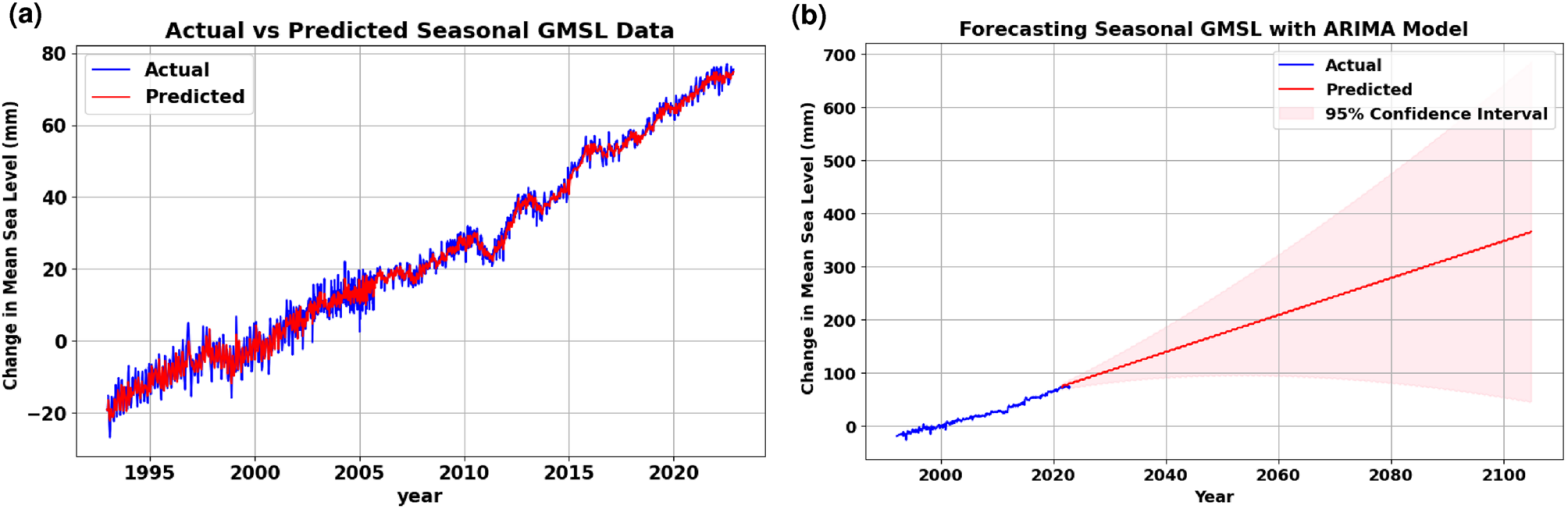
Results of applying ARIMA model on seasonal GMSL data, where (**a**) shows the prediction results against the actual data, and (**b**) shows the long-term forecasting results of the model. (a) Actual vs. predicted seasonal GMSL. (b) Long-term Forecasting of seasonal GMSL.

The outcome of this model yielded a root mean square error (RMSE) of 1.96 mm. Figure 2a and b show the obtained prediction and forecasting results of this model, respectively.

**Figure 3:**
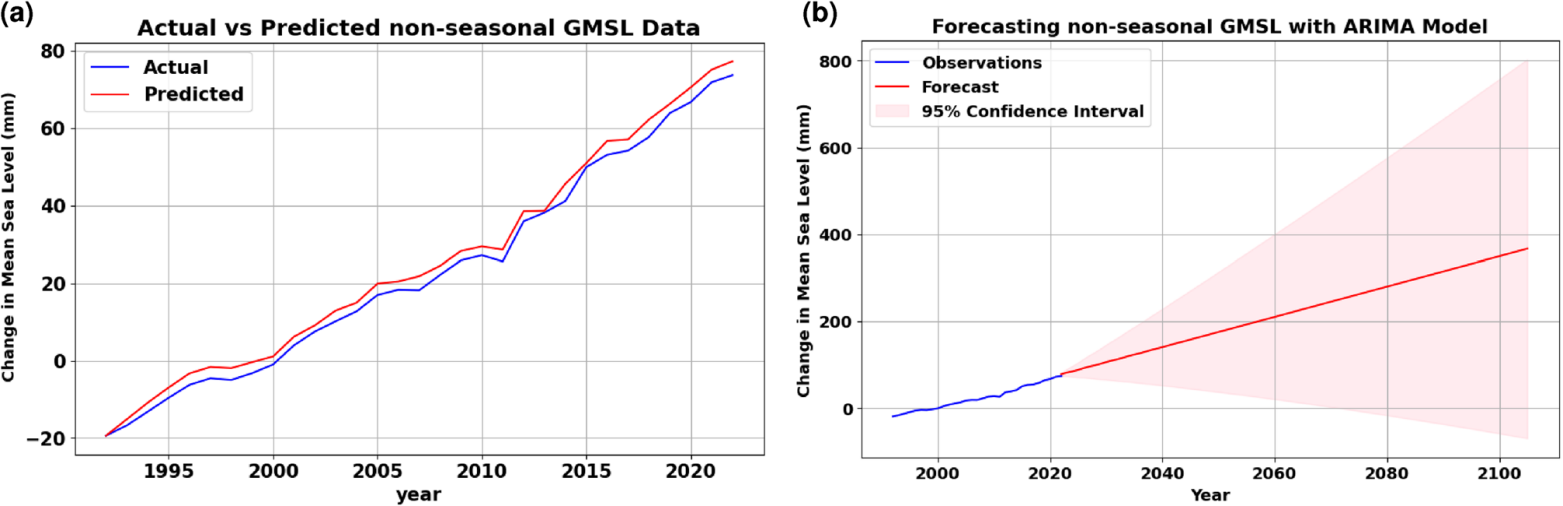
Results of applying ARIMA model on non-seasonal GMSL data, where (**a**) shows the prediction results against the actual data, and (**b**) shows the long-term forecasting results of the model applying. (a) Prediction of non-seasonal GMSL data using ARIMA. (b) Long-term forecasting of non-seasonal data using ARIMA model.

**Figure 4:**
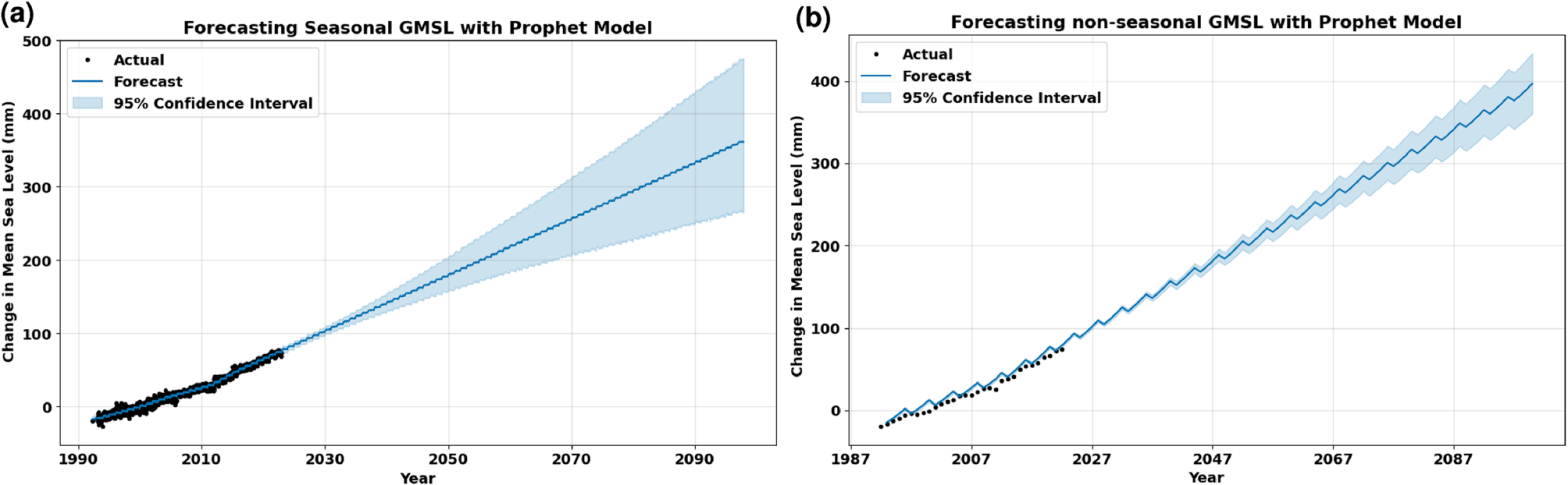
Results of applying Prophet model on GMSl data, where (**a**) shows the prediction results against the actual data, and (**b**) shows the long-term forecasting results of the model applying. (a) Long-term forecasting of seasonal data. (b) Long-term forecasting of non-seasonal data.

The second scenario eliminated the seasonality trend from the time series. The removal of seasonality trends from the time series resulted in a higher RMSE value of 2.47 mm. This adjustment underscores the importance of accounting for seasonal variations in the data, as reflected in the RMSE metric, which measures the disparity between observed and predicted values. Both scenarios were tested using the walk-forward method to assess the data. Figure 3a shows the results of non-seasonal prediction of GMSL data using ARIMA (2,1,3) and Fig. 3b shows the forecasted results till the end of the century. Figure 4 shows the prediction and forecasting results of applying the prophet model on seasonal and non-seasonal GMSL data.

Despite achieving higher results using similar AR and MA conditions, the ARIMA model with seasonal data exhibited higher accuracy than the ARIMA model with non-seasonal data when it comes to long-term forecasting as illustrated in Figs. 2b and 3b. The forecasting output of the Prophet model, as shown in Fig. 4, demonstrated its ability to capture long-term seasonality, underlying patterns, and trends of both seasonal and non-seasonal time series and successfully conforming them over the extended forecasting horizon. This nuanced capacity of the Prophet model underscores its resilience and ability to grasp the underlying patterns even in non-seasonal or ambiguous data patterns, contributing to its efficacy in forecasting scenarios. Despite having higher RMSE than ARIMA model outputs, the Prophet model exhibits higher confidence levels of short-term forecasting results than ARIMA. However, all models successfully demonstrated the anticipated positive trend. Overall, both ARIMA and Prophet models demonstrated strong performance in forecasting future sea levels on seasonal and non-seasonal data.

The forecasting scenarios involving non-seasonal data revealed a realistic depiction of the changing trend in the long-term analysis. This highlights the necessity for a proper assessment of the role of seasonality when forecasting long-term trends using autoregressive models. Table 1 compares the tested model for univariate time series analysis.

**Table 1:** Comparison of models used in univariate GMSL time series analysis.

Model	RMSE (mm)
ARIMA applied on seasonal GMSL with AR:2, MA:3, I:1 and 12 months seasonality	1.96
ARIMA applied on non-seasonal GMSL with AR:2, MA:3, I:1	2.47
Prophet model applied on seasonal GMSL	3.36
Prophet model applied on non-seasonal GMSL	9.34

Additional experiments were conducted to compare the output of the univariate models with long-short term memory (LSTM) resulting in an RMSE of 3.07 mm and good predictions for the short-term horizon. However, the LSTM model here demonstrated poor performance and couldn’t capture the trend with an extended period due to the limited size of the training dataset. Other studies, to the best of our knowledge, that utilized deep learning, particularly LSTM, in sea level analysis, focused only on short-term forecasting. Additionally, those models may experience overfitting depending on the training data set characteristics and may not generalize well with external data sets for validations^25^. Therefore, further analysis and comparison with LSTM and other deep learning models were excluded in this study as our forecasting analysis was focused on long-term forecasting (i.e. end of the current century).

### Multivariate time series analysis

In order to investigate the initial findings derived from the correlation matrix and analyze the mutual influence among factors, the ‘$select\_order$’ function from the statsmodels^26^ library in Python was utilized. This function is specifically designed for selecting the optimum lag order in a VAR model based on the Akaike information criterion (AIC).

**Figure 5:**
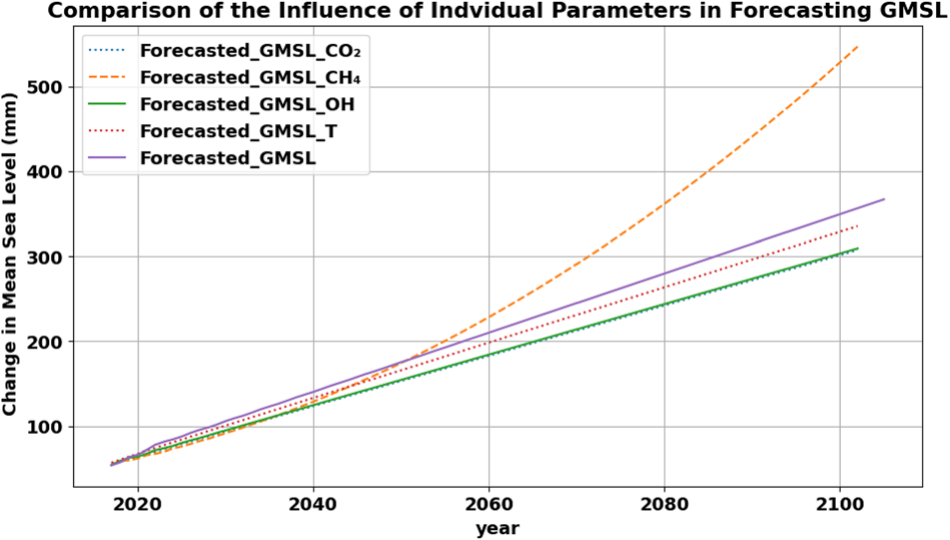
Comparison of the GMSL forecasting results in accordance to historical observations (purple), and under the influence of CO_2_ emissions (blue), CH_4_ (orange), Ocean Heat (green), and Global Temperature Change (red).

The one-on-one influence of each factor on GMSL was tested using the VAR model with the lag order chosen to minimize the AIC. A thorough testing process was conducted to ensure the robustness of the model and its ability to capture the dynamics of the time series. Figure 5 shows a comparison of the forecasting output of GMSL under the influence of each Factor.

**Figure 6:**
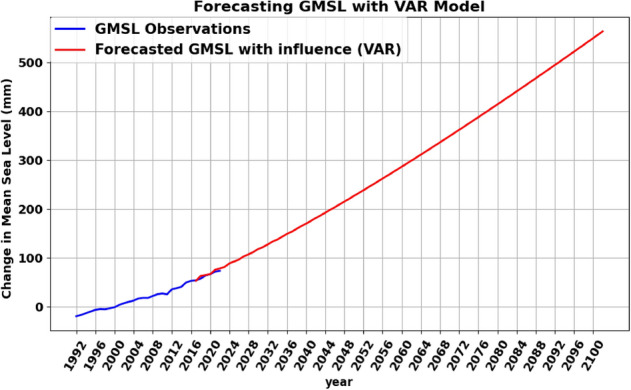
Results of long-term forecasting of GMSL using VAR model, where the cumulative influence of all selected contributing factors is applied.

**Figure 7:**
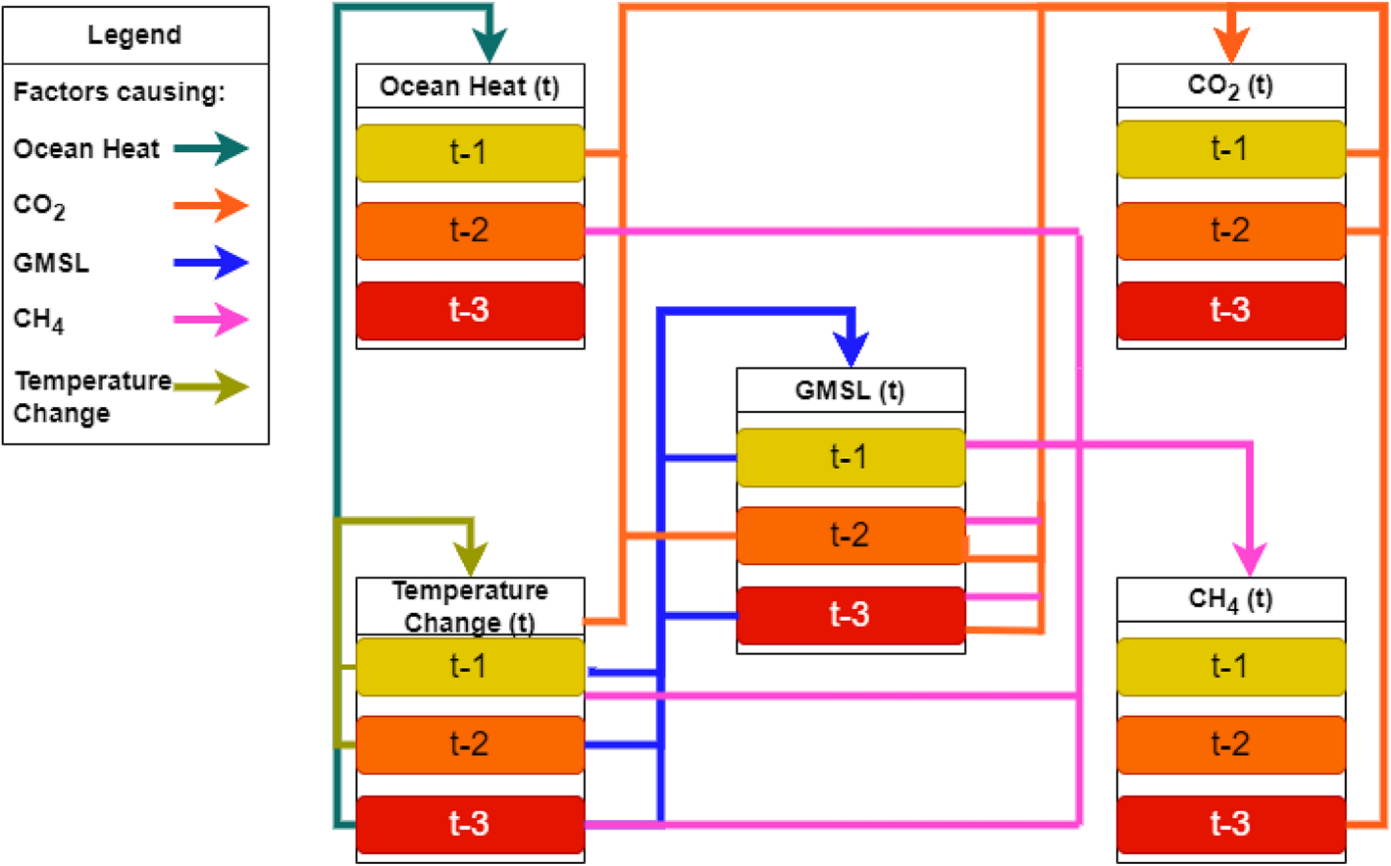
Visual illustration of the interdependent forecasting relationship among variables, where the past values of significant variables are used to predict each variable at a time t.

The group-to-one testing scenarios via the VAR (3) model were implemented to assess the influence of all contributing factors on GMSL, as illustrated in Fig. 6 where the cumulative effect of all contributing factors on GMSL is evident. To unveil the complex interrelations between the factors and how they mutually influence each other, the equations were formulated with only values and coefficients that reject the null hypothesis ($p<0.05$). The following functions present the interrelation among the contributing factors, highlighting the variables that have a significant impact only in forming the Eqs. (2), (3), (4), (5), and (6). Figure 7 visually illustrated the interrelation among variables and their previous lags in the equations. It showed that at a given time t, future changes in CO_2_ can only be attributed to previous observations of the other factors. However, previous observations of CO_2_ do not play any role in forecasting any of the other variables. The results also showed that changes in GMSL are influenced by previous sea level and air temperature changes. Similarly, previous temperature values influence its future levels and changes in ocean heat. The relationship between CH_4_ and CO_2_ outputs are unidirectional, where changes in their future values indicate changes in GMSL. However, neither variable directly influences changes in GMSL, as per Eq. (2). This mathematical logic contradicts the notion that these variables contribute directly to changes in GMSL; nonetheless, changes in their emissions can be used to monitor changes in GMSL.

2 $$\begin{aligned} \displaystyle GMSL_t = 23.095+0.090\ T_{(t-1)}+ 0.250\ GMSL_{(t-1)}+0.068\ T_{(t-2)}+0.272 \ GMSL_{(t-3)}+ 0.074\ T_{(t-3)} \end{aligned}$$

3 $$\begin{aligned} \begin{aligned} \displaystyle CO2_{t}&= 383.977+0.109\ CO2_{{(t-1)}}+ 0.108\ OH_{(t-1)}+0.753\ T _{(t-1)}+0.212\ GMSL_{(t-2)}+ 0.122\ CO2_{{(t-2)}} \\ {}&\quad + 0.753\ T _{(t-2)}+ 0.218\ GMSL_{(t-3)}-0.072\ CH4_{{(t-3)}}+0.576\ T _{(t-3)} \end{aligned} \end{aligned}$$

4 $$\begin{aligned} \begin{aligned} \displaystyle CH4_{t}&= 1799.113+0.201\ GMSL_{(t-1)}-\ 0.043\ T_{(t-1)}+0.457\ GMSL_{(t-2)}+0.162\ OH_{(t-2)}+ 0.536\ GMSL_{(t-3)} \\&\quad +0.185\ T_{(t-3)} \end{aligned} \end{aligned}$$

5 $$\begin{aligned} \displaystyle OH_t = 9.497+0.189\ T _{(t-1)}+ 0.163\ T_{(t-2)}+0.134\ T_{(t-3)} \end{aligned}$$

6 $$\begin{aligned} \displaystyle T _t = 0.633+0.061\ T _{(t-1)}+ 0.046\ T_{(t-2)} \end{aligned}$$

**Figure 8:**
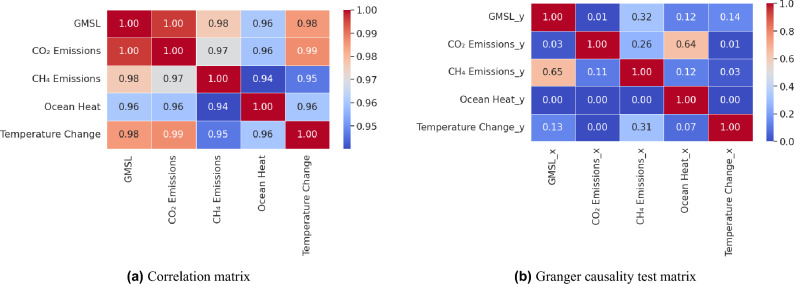
Test matrices of the data attributes that were used in this study where (**a**) represent numerical correlation matrix and (**b**) represent Granger’s causality test where values below threshold (i.e. 0.05) indicate that there is a strong influence in horizontal variable causing the vertical variable.

While all the forecasting time series exhibited linear increase patterns for the long-term as shown in Fig. 5, CH_4_ emissions influenced the forecasting results into an exponential increase over a long-term period. Figure 8a shows that there is a high numerical correlation between GMSL and CH_4_, however Granger’s test results, as can be observed in Fig. 8b, did not indicate a strong correlation in predicting those two variables in respect to each other. This can be attributed to the fact that there is a complex underlying correlation among the climatic attributes and multiple tests are required to validate the correlations among interrelated variables. This impact of CH_4_ also suggests that there will be an accelerating increase in sea levels in the absence of proper mitigation and adaptation measures that limit its emissions. Table 2 shows RMSE results obtained by using VAR model to assess the one-to-one influence of the different parameters on GMSL. On the contrary, the analysis revealed that the cumulative influence of the variables differs from scientific expectations. Equations (2) to (6) demonstrate that future changes in CO_2_ and CH_4_ are affected by alterations in GMSL, while the opposite is not true. Therefore, changes in CO_2_ and CH_4_ indicate changes in GMSL.

**Table 2:** Comparison of the influence of individual factors on GMSL forecasting.

Contributing factor	RMSE (mm)
GMSL $\rightarrow$ GMSL	2.13
CO_2_ $\rightarrow$ GMSL	10.64
CH_4_ $\rightarrow$ GMSL	9.67
Ocean heat $\rightarrow$ GMSL	11.45
Global temperature change $\rightarrow$ GMSL	13.86

### Regional mean sea level: the Arabian Gulf

**Figure 9:**
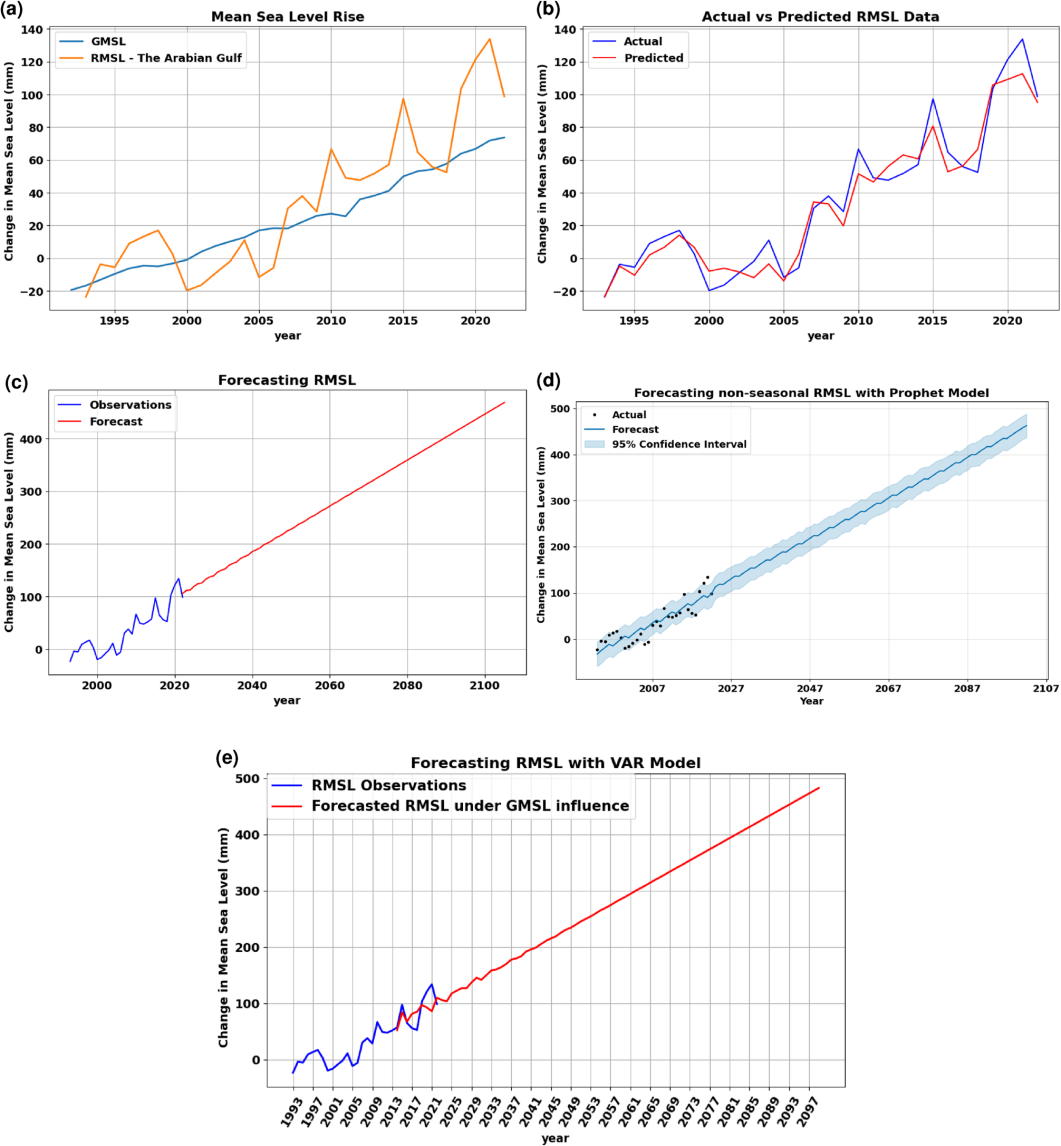
Illustration of the analysis results conducted on RMSL time series data. (**a**) GMSL vs. RMSL (the Arabian Gulf), (**b**) Predicting RMSL time series with ARIMA (2,1,3) model, (**c**) Forecasting non-seasonal RMSL with ARIMA model, (**d**) Forecasting results of RMSL with Prophet model, and (**e**) Forecasting RMSL under the influence of GMSL with VAR model.

The non-seasonal RMSL time series of the Arabian Gulf exhibited more fluctuation patterns than GMSL as shown in Fig. 9a. Generally, the values of RMSL were higher than GMSL by more than 40% while maintaining the increasing trend. This higher fluctuation pattern was used to provide additional insights into the performance of the models in such scenarios. The fluctuation pattern of this time series was captured with ARIMA (2,1,3). However, it resulted in a respectively high RMSE value of 18.29 mm. This indicated a higher deviation of the actual data as shown in Fig. 9b. The fluctuation in RMSL time series with and without the influence of predictors has resulted in a non-linear pattern in short-term period forecasting that gradually transitioned to a more linear pattern with the extension of time as shown in Fig. 9c. This is attributed to the dependency of data on previous observations. Conversely, the Prophet model, as shown in Fig. 9d, demonstrated fluctuations patterns over long-term forecasting. Similar behavior was also observed in RMSL forecasting under the influence of GMSL, which resulted in smaller deviations of forecasted results as shown in Fig. 9e. Despite being less accurate in capturing the underlying pattern, the Prophet model possesses the capability of conforming to the pattern and maintaining it alongside the increasing trend over extended time periods. Overall, across all forecasting models on both seasonal and non-seasonal data, the confidence level interval in the long-term forecasting results was higher (narrower) with the Prophet model than with ARIMA, suggesting higher reliability in such scenarios.

## Conclusion

In conclusion, this study examined the application of the selected time series analysis models in forecasting sea level. ARIMA model was applied to both seasonal and non-seasonal time series. Additionally, the forecasting capabilities of Facebook’s Prophet model, known for its successful application in weather data prediction^19^, were explored in predicting and forecasting sea levels. Both the ARIMA and Prophet models showed good performance, with the latter having the ability to show patterns and trends over long-term forecasting periods with higher confidence intervals while ARIMA resulted in linear forecasting.

The impact of various climatic-climatic factors contributing to GMSL, such as ocean heat, temperature, and CO_2_ and CH_4_ emissions, was also investigated in this study. The influence of individual variables and the cumulative impact of all variables on GMSL were examined using a VAR model, which provided an analysis of the complex underlying interrelationships among the variables and assessed the significance of their influence in predicting each other. The analysis results revealed a distinction between scientific and empirical relationships among the variables. Although CO_2_ acts as an indicator of changes in GMSL and temperature, alterations in GMSL and temperature do not necessarily affect future changes in CO_2_ or CH_4_ statistically.

Furthermore, this study examined the variation between GMSL and a selected RMSL study area, the Arabian Gulf. Analysis of the non-seasonal RMSL data from the Arabian Gulf revealed a distinct fluctuation pattern. This allowed for an assessment of the conclusions drawn from the GMSL analysis regarding the performance of the ARIMA and Prophet models when dealing with datasets of different natures, such as unstructured and complex fluctuations. The results highlighted the advantage of autoregressive models over the Prophet model.

## Methods

### Autoregressive models

ARIMA is a widely used time series forecasting model that combines AR and MA components with differencing to handle non-stationary data. ARIMA is capable of capturing and predicting temporal patterns in univariate time series data. The AR component accounts for the linear relationship between an observation and its past values, while the MA component considers the influence of past forecast errors. The “integrated” part signifies the differencing step applied to achieve stationarity, making the model applicable to a broader range of time series. ARIMA components include AR (AutoRegressive), I (Integrated), and MA (Moving Average), represented by parameters ‘p’, ‘d’, and ‘q’ respectively. These parameters determine the corresponding component value for fitting the model. ARIMA model is suitable for analyzing climatic data analyses based on prediction of the temporal patterns^27^. The general formula for the ARIMA model is:

7 $$\begin{aligned} \displaystyle y_t = c+ \phi _1 y_{t-1}+ \phi _2 y_{t-2}+ \cdots + \phi _p y_{t-p}+ \varepsilon _t \end{aligned}$$

where ${y_t}$ is the time series being modeled. *c* is the constant term (mean of the series). ${\phi _1,\phi _2,\dots , \phi _p}$ are AR parameters for lagged values. ${\varepsilon _t}$ is the white noise error term at time t.

While ARIMA models are used on univariate time series, VAR is a statistical modeling technique that is used to analyze the dynamic interrelationships among multiple time series variables. Unlike univariate time series models, VAR enables the examination of the mutual dependencies between different variables where each variable is represented as a linear combination of its past values as well as the past values of other variables in the system under the assumption that all variables in the system are interrelated and influence each other. VAR models are often used to understand the underlying complex relationship and temporal dependencies among the variables in multivariate time series data. The main component of VAR model is the lag order (p)^27^. The general form of a VAR(p) model is given by:

8 $$\begin{aligned} \displaystyle Y_t = \alpha +\sum _{i=1}^{p}A_i \ Y_{t-i}+\varepsilon _t \end{aligned}$$

where ${Y_t}$ is a vector of endogenous variables at time t; ${\alpha }$ is a constant vector; ${A_i}$ are coefficient matrices for the lagged values of the endogenous variables up to lag p; ${\varepsilon _t}$ is a vector of error term.

The Augmented Dickey–Fuller (ADF) test is a statistical test used to assess the stationarity of a time series. This test determines whether a given time series is stationary by checking for its unit root. The null hypothesis is tested against a p threshold value (often 0.05) such that the rejection is based on whether the value is less than the threshold value indicating that it is stationary. Ensuring that the time series is stationary before fitting the model is crucial in obtaining reliable and accurate forecasting results. One method of transforming the time series to stationary is differencing. The ‘I’ parameter is determined by the order of differencing which renders the data into stationary. The ‘q’ and ‘p’ parameters can be determined by the Autocorrelation Function (ACF), “that measures the linear relationship between lagged values of a time series”, and Partial Auto Correlation Function (PACF), which measures this relation in the absence of the effect of intermediate lags, respectively^27^.

### Facebook’s prophet model

The Facebook Prophet is a forecasting model designed to handle time series that include seasonality, multiple trend components, and even holiday components. This model was developed by Facebook and can capture complex seasonal patterns and handle missing data, which makes it suitable for a wide variety of real-world applications, including climate forecasting^27^. The general equation for the prophet model is:

9 $$\begin{aligned} \displaystyle y(t) = g(t)+s(t)+h(t)+{\varepsilon _t} \end{aligned}$$

where *y*(*t*) represents the observed value at time t; *g*(*t*) is the trend component modeling the non-periodic changes over time; *s*(*t*) represents the seasonal component capturing periodic changes; *h*(*t*) denotes the effects of holidays and special events; ${\varepsilon _t}$ is the error term representing any idiosyncratic noise or unexpected fluctuations.

### Data

Tide gauges and satellite altimetry are the main sources for monitoring sea levels. Tide gauges provide detailed, long-term records of sea level at specific locations and valuable historical data for studying sea level changes. Despite their precision in coastal monitoring, tide gauges are often sparse, unevenly distributed, and provide “relative” measurements of sea level to the ground. This relative measurement is influenced by land subsidence and requires the estimation of vertical land motion to determine the absolute sea level change. Conversely, Satellite altimetry provides “absolute” sea level rise measurement with high precision and enables continuous monitoring of the sea level variations^28^.

The data utilized in this research were obtained from two main sources: the National Oceanic and Climatic Administration (NOAA) and the National Aeronautics and Space Administration (NASA). Sea level data were obtained from NOAA where measurements were conducted using various radar satellite altimeters: TOPEX/Poseidon (T/P), Jason-1, Jason-2, and Jason-3^29^. Global sea level data were processed to produce the mean average of the annual and monthly data. The Arabian Gulf was selected as the focus area for RMSL analysis due to its distinction as one of the world’s warmest water bodies. The increasing coastal populations in Gulf basin countries increased the human activities and the operation of seawater desalination plants, which influence variations of sea levels^30^. Furthermore, the tidal wave due to wind effect significantly affected the seasonal sea level variations^31^. Due to the ambiguous timestamps of seasonal RMSL data, only non-seasonal RMSL data were considered in this study.

**Figure 10:**
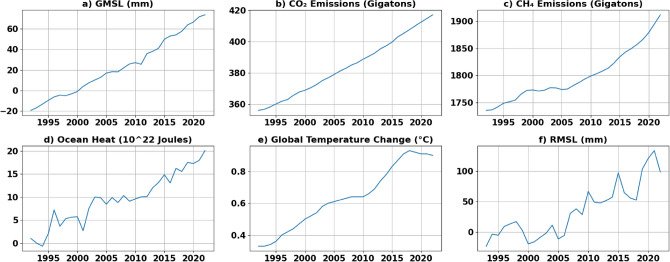
The used time series data of (**a**) GMSL in mm (**b**) global CO_2_ emissions in gigatons (**c**) global CH_4_ emissions in gigatons (**d**) ocean heat measured in 10$^{22}$ J (**e**) global change in temperature in Celsius (**f**) regional mean sea level (RMSL).

Additionally, GHG data, specifically CO_2_^32^ and CH_4_^33^, were obtained from NOAA and presented in annual format with their associated uncertainties spanning the years from 1992 till 2022. The annual data for ocean heat (ocean warming) and global temperature change were obtained from NASA^34^ for the years between 1992 and 2022. To unify the analysis, seasonality was removed from sea level data. Figure 10 illustrates the time series data used in this study. All data used in this study are publicly available.

Given that GMSL is scientifically influenced by the selected contributing factors, the pairwise correlations among their corresponding time series were investigated using the correlation matrix. Figure 8a illustrates that all the selected factors exhibit strong numerical correlation, with the most substantial correlations observed between CO_2_ emissions and GMSL, followed by CO_2_ emissions and global temperature change. The initial findings from this correlation analysis suggest a significant influence of these factors on GMSL.

### Models development

The preprocessing of data and model training were carried out on a Jupyter notebook^35^ running on Python v3.10.12^36^. Various Python libraries and packages were utilized, including Statsmodel^26^ v0.14.2, Scikit-Learn^37^ v1.2.2, and Pandas^38^ v2.0.3. The Matplotlib^39^ library was used to generate graphs of data and the obtained results while Draw.io tool^40^ v24.4.13 was used to generate the illustrative diagrams (i.e. Figs. 1 and 7).

**Figure 11:**
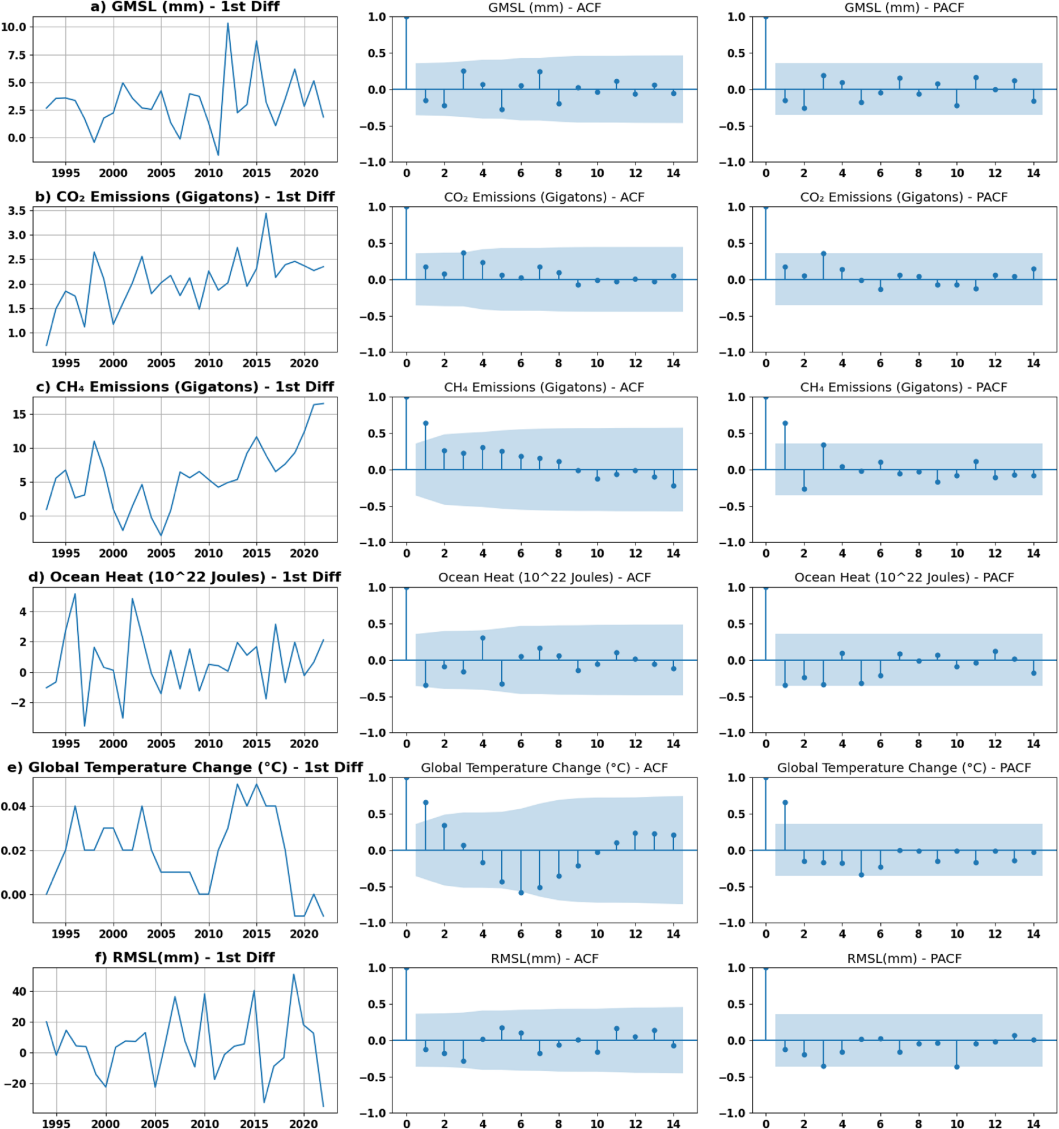
Stationary plot, ACF and PACF graphs generated for each time series with 1st order differencing.

**Figure 12:**
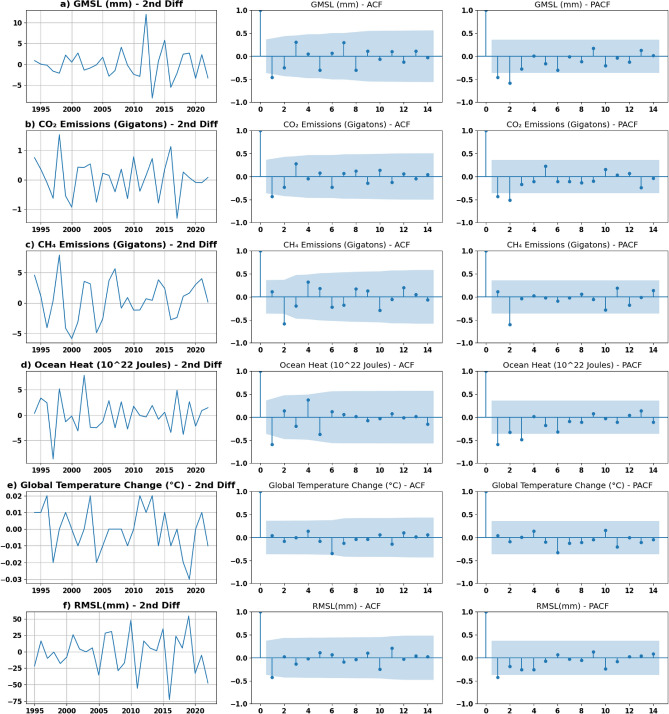
Stationary plot, ACF and PACF graphs generated for each time series with 2nd order differencing.

As the numerical correlation was proved in Fig. 8a, additional examining for the correlation among the variables was tested using the Granger causality test. Granger causality is a statistical test that examines whether the past values of a specific time series can significantly influence the prediction of another time series. Figure 8b illustrates the matrix obtained from running Granger’s causality statistical correlation test over a maximum of 3 lags, represented by the lowest obtained *p* values. Upon testing the values in the matrix against the significance threshold of 0.05 (i.e. *p* values less than 0.05 significantly influence the prediction of the other value), it becomes evident that CO_2_ plays a significant role in causing GMSL. Furthermore, changes in global temperature contribute significantly to changes in CO_2_, CH_4_, and ocean heat. However, this influence was not found significant in the context of GMSL. This matrix also demonstrates unidirectional relations among some variables. For instance, changes in GMSL can be used as an indicator of changes in ocean heat, however, the opposite relation is not necessarily true. Figures 11 and 12 illustrate the differenced time series with 1st and 2nd differencing orders alongside their ACF and PACF figures. Those figures were used to validate the ‘p’, ‘d’, ‘q’ parameters selection. The performance of all models in this study was evaluated using RMSE.

## Data Availability

The datasets used in this study are publicly available. Altimetry data of sea levels are provided by the NOAA Laboratory for Satellite Altimetry at https://www.star.nesdis.noaa.gov/socd/lsa/SeaLevelRise/LSA_SLR_timeseries.php. GHG data are available at https://gml.noaa.gov/ccgg/trends/gl_data.html. Ocean heat and global temperature change are available at https://climate.nasa.gov/vital-signs/ocean-warming/, and https://climate.nasa.gov/vital-signs/global-temperature/, respectively. Data processing and analysis results were included in this article.

## References

[1] Nicholls, R. *et al.* Constructing sea-level scenarios for impact and adaptation assessment of coastal areas: A guidance document. In *Supporting Material, Intergovernmental Panel on Climate Change Task Group on Data and Scenario support for Impact and Climate Analysis (TGICA)*, vol. 47 (2011).

[2] Elneel L, Zitouni MS, Mukhtar H, Galli P, Al-Ahmad H (2024). Exploring key aspects of sea level rise and their implications: An overview. Water.

[3] Noor NM, Abdul Maulud KN (2022). Coastal vulnerability: A brief review on integrated assessment in southeast Asia. J. Mar. Sci. Eng..

[4] Oppenheimer M, Pörtner HO, Roberts DC, Masson-Delmotte V, Zhai P, Tignor M, Poloczanska E, Weyer NM (2022). Sea level rise and implications for low-lying islands, coasts and communities. The Ocean and Cryosphere in a Changing Climate.

[5] Faridatunnisa, M. & Heliani, L. S. Study of sea level rise using tide gauge data year 1996 to 2015 at semarang and prigi stations. In *2018 4th International Conference on Science and Technology (ICST)*, 1–4. 10.1109/ICSTC.2018.8528668 (IEEE, 2018).

[6] Tabassum, A., Rabbani, M. & Omar, S. B. An approach to study on time series components and by using them to enumerate the height of sea level alteration for both global mean sea level (GMSL) and Bay of Bengal (BOB). In *2019 IEEE International Conference on Electrical, Computer and Communication Technologies (ICECCT)*, 1–7. 10.1109/ICECCT.2019.8869397 (IEEE, 2019).

[7] Chung J, Tong G, Chao J, Zhu W (2021). Path analysis of sea-level rise and its impact. Stats.

[8] Hassan, K. M. A., Haque, M. A. & Ahmed, S. Comparative study of forecasting global mean sea level rising using machine learning. In *2021 International Conference on Electronics, Communications and Information Technology (ICECIT)*, 1–4. 10.1109/ICECIT54077.2021.9641339 (IEEE, 2021).

[9] Krishnamurthy, V. N. D., Degadwala, S. & Vyas, D. Forecasting future sea level rise: A data-driven approach using climate analysis. In *2023 2nd International Conference on Edge Computing and Applications (ICECAA)*, 646–651. 10.1109/ICECAA58104.2023.10212399 (IEEE, 2023).

[10] Elneel, L., Zitouni, M. S., Mukhtar, H. & Al-Ahmad, H. Forecasting global mean sea level rise using autoregressive models. In *2023 30th IEEE International Conference on Electronics, Circuits and Systems (ICECS)*, 1–4. 10.1109/ICECS58634.2023.10382721 (IEEE, 2023).

[11] Tur R, Tas E, Haghighi AT, Mehr AD (2021). Sea level prediction using machine learning. Water.

[12] Balogun AL, Adebisi N (2021). Sea level prediction using ARIMA, SVR and LSTM neural network: Assessing the impact of ensemble ocean-atmospheric processes on models’ accuracy. Geomat. Nat. Hazards Risk.

[13] Adebisi N, Balogun A-L (2022). A deep-learning model for national scale modelling and mapping of sea level rise in Malaysia: The past, present, and future. Geocarto Int..

[14] Ahmed AN (2022). Water level prediction using various machine learning algorithms: A case study of durian Tunggal river, Malaysia. Eng. Appl. Comput. Fluid Mech..

[15] Das N (2022). Time series forecasting of temperature and turbidity due to global warming in river ganga at and around Varanasi, India. Environ. Monit. Assess..

[16] Elseidi M (2023). A hybrid Facebook prophet-ARIMA framework for forecasting high-frequency temperature data. Model. Earth Syst. Environ..

[17] Haris, M. D., Adytia, D. & Ramadhan, A. W. Air temperature forecasting with long short-term memory and prophet: A case study of Jakarta, Indonesia. In *2022 International Conference on Data Science and Its Applications (ICoDSA)*, 251–256. 10.1109/ICoDSA55874.2022.9862869 (IEEE, 2022).

[18] Thiyagarajan, K., Kodagoda, S., Ulapane, N. & Prasad, M. A temporal forecasting driven approach using Facebook’s prophet method for anomaly detection in sewer air temperature sensor system. In *2020 15th IEEE Conference on Industrial Electronics and Applications (ICIEA)*, 25–30. 10.1109/ICIEA48937.2020.9248142 (IEEE, 2020).

[19] Toharudin T (2023). Employing long short-term memory and Facebook prophet model in air temperature forecasting. Commun. Stat. Simul. Comput..

[20] Basak A, Rahman AS, Das J, Hosono T, Kisi O (2022). Drought forecasting using the prophet model in a semi-arid climate region of western India. Hydrol. Sci. J..

[21] Fronzi D (2024). Towards groundwater-level prediction using prophet forecasting method by exploiting a high-resolution hydrogeological monitoring system. Water.

[22] Lincoln S (2021). A regional review of marine and coastal impacts of climate change on the ROPME sea area. Sustainability.

[23] Hereher ME (2020). Assessment of climate change impacts on sea surface temperatures and sea level rise-the Arabian Gulf. Climate.

[24] van den Bosch, M. *Five Climate Challenges the Gulf States Might Not Have Time to Solve*. https://agsiw.org/five-climate-challenges-the-gulf-states-might-not-have-time-to-solve/ (2023).

[25] Kinoyama, R., Perez, E. A. M. & Iba, H. Preventing overfitting of LSTMS using ant colony optimization. In *2021 10th International Congress on Advanced Applied Informatics (IIAI-AAI)*, 343–350. 10.1109/IIAI-AAI53430.2021.00061 (IEEE, 2021).

[26] Statsmodels. https://www.statsmodels.org/.

[27] Hyndman RJ, Athanasopoulos G (2018). Forecasting: Principles and Practice.

[28] Cazenave A, Cozannet GL (2014). Sea level rise and its coastal impacts. Earth’s Future.

[29] NOAA. *Laboratory for Satellite Altimetry/Sea Level Rise*. https://www.star.nesdis.noaa.gov/socd/lsa/SeaLevelRise/LSA_SLR_timeseries.php.

[30] Al-Maamary HM, Kazem HA, Chaichan MT (2017). Climate change: The game changer in the gulf cooperation council region. Renew. Sustain. Energy Rev..

[31] Chow AC, Sun J (2022). Combining sea level rise inundation impacts, tidal flooding and extreme wind events along the Abu Dhabi coastline. Hydrology.

[32] Lan, X., Tans, P. & Thoning, K. *Trends in Globally-Averaged Co2 Determined from NOAA Global Monitoring Laboratory Measurements*. 10.15138/9N0H-ZH07 (2023).

[33] Thoning, K., Dlugokencky, E. & Lan, X. *Trends in Globally-Averaged CH4, N2O, and SF6* (2022). 10.15138/P8XG-AA10.

[34] NASA. *Global Climate Change|Vital Signs of the Planet*. https://climate.nasa.gov (2024). Accessed 3 Jan 2024.

[35] Project jupyter | home. https://jupyter.org/.

[36] Python 3.10.12. https://www.python.org/downloads/release/python-31012/.

[37] scikit-learn machine learning in python. https://scikit-learn.org/stable/.

[38] Pandas. https://pandas.pydata.org/.

[39] Matplotlib: Visualization with python. https://matplotlib.org/.

[40] Draw.io. https://www.drawio.com/.

